# Fluorescence of 2-Hydroxy Chalcone Analogs with Extended Conjugation: ESIPT vs. ICT Pathways

**DOI:** 10.3390/molecules29245972

**Published:** 2024-12-18

**Authors:** Brian Corbin, Paityn Houglan, Yi Pang

**Affiliations:** Department of Chemistry, The University of Akron, Akron, OH 44325, USA

**Keywords:** chalcone, ESIPT, charge transfer, ICT, low-temperature fluorescence

## Abstract

The chalcone derivatives with hydroxy group (**2**) have been examined using low-temperature fluorescence spectroscopy. The study aimed to freeze the intramolecular charge transfer (ICT) motion in order to reveal the potential hidden transition(s) that are difficult to observe at room temperature. Although chalcone **2** revealed one emission peak at ~667 nm at room temperature, it exhibited two emission peaks (λ_em_ = 580 and 636 nm) in EtOH at liquid N_2_ temperatures (77 K). With the aid of model compound **3** with methoxy group and aluminum complex **2**-Al^3+^, attempts were made to assign these emission peaks. The results point towards the possibility of the coexistence of ICT and excited state intramolecular proton transfer (ESIPT) in the chalcone derivatives with extended conjugation.

## 1. Introduction

**Scheme 1:**
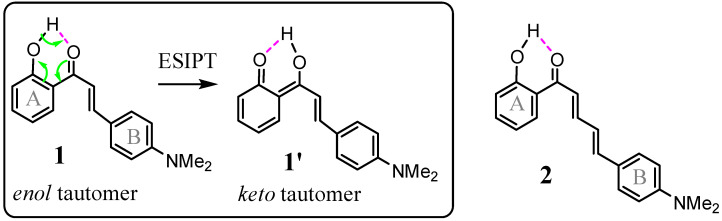
Structures of 2′-hydroxychalcones **1** and **2**, where dashed line indicate H-bonding.

As a class of natural organic compounds found in plants, chalcones have a general structure of Ar–CO–CH=CH–Ar’, making them useful precursors for the synthesis of flavonoids. This class of compounds also exhibit attractive optical properties for different applications. For example, 2′-hydroxychalcone **1** and its derivatives are known to give emission with a large Stokes’ shift (e.g., λ_abs_ ≈ 430 nm, λ_em_ ≈ 620 nm in benzene) [1]. A notable feature of **1** is that the phenol group on the A ring can form the intramolecular hydrogen bonding (Scheme 1), which can undergo excited state intramolecular proton transfer (ESIPT) upon irradiation [2,3]. In addition, chalcone **1** also includes an amine group (NMe_2_) as an electron donor and a carbonyl group as an acceptor, enabling the intramolecular charge transfer (ICT). Coupling of ESIPT with ICT has led to various applications of **1**, including the design of molecular probes for biothiols [4,5], selective detection of human serum albumin (HSA) [6], detection of Al^3+^ and Cu^2+^ Ions [7,8], and solid state near-infrared (NIR) emissive materials [9].

As a structurally similar compound to **1**, chalcone **2** includes an extended π-conjugation by inserting one additional C=C double bond between the A and B rings. Although ESIPT in **1** is well demonstrated, experimental evidence for possible ESIPT in **2** is still lacking in our knowledge. It is expected that strong ICT interaction would be present between a strong electronic donor (i.e., -NMe_2_) and acceptor (i.e., C=O) groups in **2**. Recently, Abou-Zied et al. [10] suggested that chalcone **2** might lack ESIPT due to a more efficient ICT that involves the strong electron-donating -NMe_2_ substituent on the B-ring. However, it remains a fundamental question why the ESIPT path is no longer competitive in **2**, even though it has nearly the same intramolecular hydrogen bonding environment as present in **1**. Further study is apparently necessary in order to seek the answer. It should be noted that the steady state fluorescence of chalcone **2** exhibits only one emission band at room temperature, which makes it difficult to differentiate the potential ESIPT emission from ICT signals.

Low-temperature fluorescence is known to be a useful method to provide information about the extent of ICT and ESIPT pathways. Among them, the ICT can operate over a relatively long distance along the pathway of donor–acceptor interaction, as shown from the structure of **1** (from the donor -NMe_2_ to the acceptor C=O). Such long-range interaction could be deterred or minimized when a fluorescent molecule is frozen in a solvent matrix under liquid N_2_ temperature (e.g., −189 °C), due to restriction on the molecular motion and bond changes. In contrast, the ESIPT process happens in a relatively localized environment. In other words, some molecular reorganization in the excited state, such as twisted intramolecular charge transfer (TICT) that requires a large change in atomic positions, could be eliminated in the frozen solvent matrix, thereby simplifying the spectra for analysis. The use of low-temperature fluorescence for the potential characterization of ESIPT and ICT has been demonstrated in a spectroscopic study for flavonoid dyes by our group [11].

**Scheme 2:**
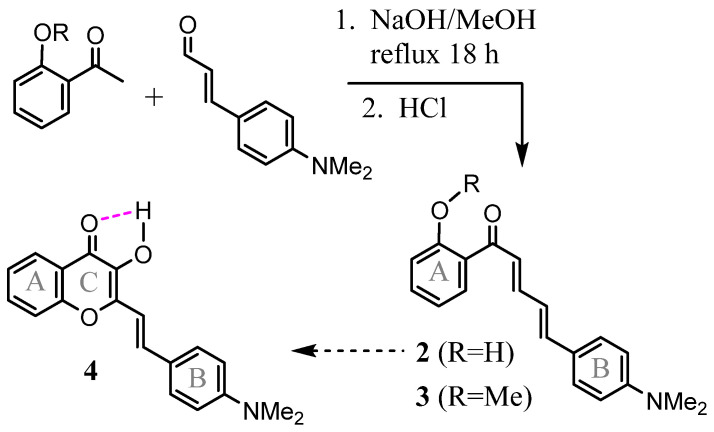
Synthetic route of chalcones **2** and **3**.

Chalcone **2** is a necessary intermediate for the synthesis of flavonoid-based red-emitting compound **4**, which has been shown to be useful for imaging of intracellular metal ions [12] and thiols [13]. In the previous study [12], we have reported the one-pot synthesis of **4** (with λ_max_ ≈ 438 nm, λ_em_ ≈ 613 nm and ϕ_fl_ ≈ 0.24 in EtOH), without isolation of chalcone intermediate **2** (Scheme 2). In addition to its bright fluorescence, flavonoid **4** also exhibited a selective response toward Al^3+^ cation, forming the metal complex **4**-Al^3+^ that generates a large spectral shift (λ_max_ ≈ 507 nm and λ_em_ ≈ 658 nm). The metal complex formation is likely accomplished by using the carbonyl (C=O) and hydroxy (OH) groups on the C ring of **4**. In an effort to gain further understanding of the optical properties of **4**, we decided to visit the synthesis and low-temperature fluorescence study of chalcone **2**. The low-temperature fluorescence of **2** could shed some light on the competitive ESIPT and ICT processes. To aid the spectroscopic study, model compound **3** was also synthesized, where the hydroxy group was replaced with a methoxy group. The switch from -OH to -OMe on the A-ring should retain ICT while eliminating the proton transfer. The methoxy group in compound **3** maintains the electron-donating ability as the phenol in **2,** which should provide a more accurate analysis versus complete removal of the hydroxy group to eliminate ESIPT. In addition, chalcone **2** could also serve as a proper ligand for Al^3+^ binding, since both **4** and **2** have the hydroxy (-OH) and carbonyl (C=O) groups next to each other, but with a different arrangement of chelating groups. Since the binding of Al^3+^ cation on **2** could induce stronger ICT interaction, in comparison with that on **4**, the study could provide some additional information on the binding kinetics and spectral shift. In this study, we disclose the fluorescence characteristics of **2** at low temperature (77 K) and the use of **2** for binding Al^3+^ cation.

## 2. Results and Discussion

### 2.1. Optical Properties

**Scheme 3:**
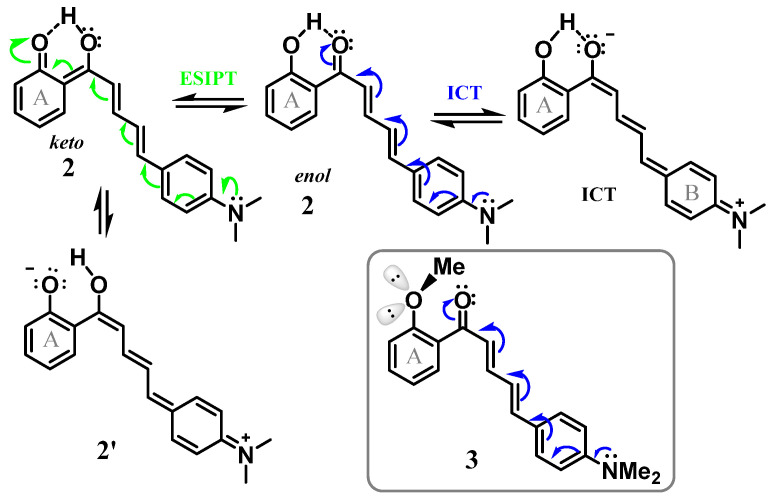
Schematic illustration of possible ESIPT and ICT interactions for chalcone **2**, in comparison with **3** with only ICT interaction. The arrows indicate possible ICT interactions.

The spectral properties of chalcones **2** and **3** were studied in various solvents, and the results are summarized in Table 1. As a proper model for **2**, the methyl group on the A-ring of **3** is forced to rotate away from the π-conjugated plane. However, such structural arrangement will not prevent the oxygen atom (attached to the A-ring) from exerting its electronic effect (Scheme 3). In a polar solvent (e.g., DCM and CH_3_CN), chalcone **2** exhibited UV-vis absorption λ_max_ = 456–461 nm (Table 1), showing notable bathochromic shift in comparison with **3** (λ_max_ = 418–424 nm in the same solvents). The observed bathochromic shift (Δλ_max_ ≈ 37–38 nm) indicated a significant impact from the intramolecular hydrogen bonding on the A-ring of **2**. The impact on the spectral shift was reduced to Δλ_max_ ≈ 25 nm in EtOH, which could be attributed to the competitive intermolecular H-bonding. It should be noted that the phenolic hydrogen of **2** was detected at 13.1 ppm in its ^1^H NMR spectra (ESI Figure S1), which is significantly deshielded and implies strong H-bonding to the carbonyl in the A-ring of **2**.

**Figure 1:**
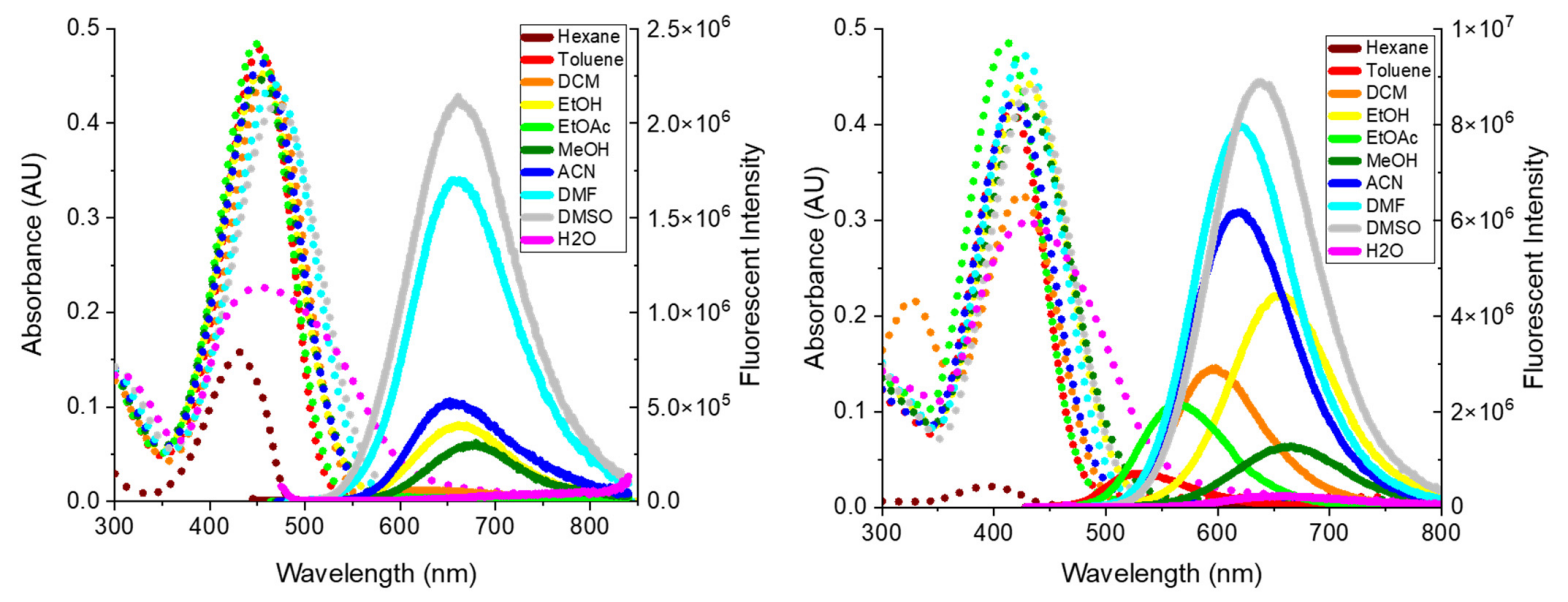
UV-vis (dotted line) and fluorescence (solid lines) of hydroxy chalcone **1** (**left**) and methoxy chalcone **2** (**right**) in various solvents at room temperature.

In a polar aprotic solvent, the fluorescence of **2** exhibited one peak in DCM (λ_em_ ≈ 628 nm) and CH_3_CN (λ_em_ ≈ 655 nm) in comparison with that of **3** at λ_em_ ≈ 580 and 601 nm in the same solvents (Figure 1 & Table 1). The fluorescence of **2** exhibited a notable fluorescence bathochromic shift (Δλ_em_ ≈ 48 and 54 nm) from that of **3**, indicating the possible contribution of ESIPT in the former, in addition to the strong ICT interaction. It should be noted that the ICT only interaction in **2** involved five double bonds, as shown by the arrows in the enol tautomer of **2** (Scheme 2). In contrast, the interaction from the C=O to -NMe_2_ group involved six double bonds (see keto tautomer of **2**). Observation of the higher Stokes shift from **2**, in all the solvents examined (except in EtOH) (Table 1), suggested that the emission may have some contribution from ESIPT. The assumption was consistent with the observed larger Stokes shift from **2** (in DCM, CH_3_CN and DMSO). In EtOH, the Stokes shift became comparable for both **2** and **3**. This could be attributed to the presence of a competitive intermolecular H-bond with solvent EtOH, which hindered the proton transfer.

The quantum yield of chalcone **3** was notably higher than **2** in all solvents tested (Table 1). For example, in CH_2_Cl_2_, the fluorescence quantum yield of chalcone **3** was Φ_fl_ ≈ 0.063, which was about 20 times higher than that of **2** (Φ_fl_ ≈ 0.003). In CDCl_3_, a solvent that has similar polarity as CH_2_Cl_2_, the ^1^H NMR of chalcone **2** detected a sharp peak at 13.1 ppm (ESI Figure S1), indicating strong intramolecular hydrogen bonding. The higher quantum yield of **3** vs. **2** could thus be attributed to the elimination of hydrogen bonding, which provides an additional vibronic pathway for nonradiative decay.

### 2.2. Low-Temperature Fluorescence

**Figure 2:**
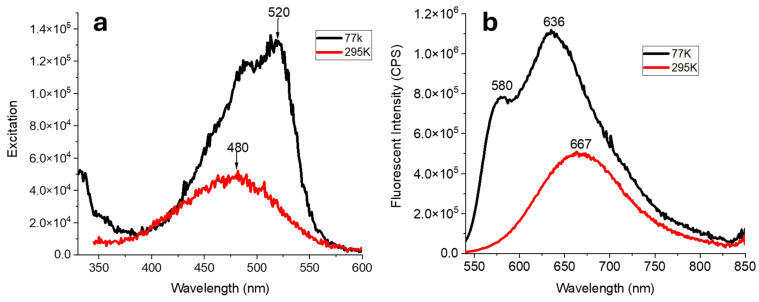
Excitation spectra (**a**) and emission spectra (**b**) of hydroxy chalcone **2** in ethanol at various temperatures.

In order to shed some light on the relationship between ESIPT and ICT in chalcone **2**, low-temperature fluorescence was performed in ethanol using liquid nitrogen (77 K) in a quartz dewar. Thus, an ethanol solution of **2** (10 μM) in a quartz tube was slowly submerged and frozen in the solvent matrix of EtOH (m.p. 159 K). At the low temperature, the excitation spectrum of frozen chalcone **2** exhibited a notable bathochromic shift from λ_ex_ = 480 nm to λ_ex_ ≈ 520 nm (or by 40 nm, Figure 2a). The fluorescence intensity of **2** was also increased by a factor of ~3 at 77 K, as the molecular motions were decreased in the frozen-solid matrix of solvent. Interestingly, chalcone **2** exhibited dual emission, showing λ_em_ at 580 and 636 nm (Figure 2b). The observed dual emission behavior from **2**, which has not been observed before (to the best of our knowledge), indicated the possible enol emission and presence of ESIPT in the 2-hydroxy chalcone. On the basis of a large spectral shift at the low temperature, the fluorescence signal at 580 nm could be attributed to ICT. It was assumed that the bond length alteration associated with the ICT process could be stopped in a frozen solvent matrix, which would result in a large spectral blue shift in emission [14].

#### 2.2.1. Low-Temperature Fluorescence of **3**

**Figure 3:**
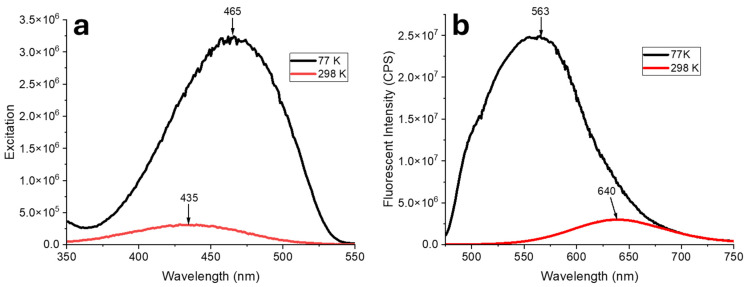
Excitation (**a**) and emission (**b**) spectra of methoxy chalcone **3** in ethanol at various temperatures.

Chalcone **3** was subjected to the same experimental conditions as **2** and the low-temperature fluorescence results are shown in Figure 3. The excitation spectrum of **3** changed similarly compared to chalcone **2**, with a spectral red shift of ~30 nm upon freezing. The fluorescence intensity of **3** was increased by a factor of ~10 at 77 K, which was notably higher than that of **2**. It should be noted that the A-ring in **3** can rotate with ease at 295 K, which provides an additional non-radiative decay pathway, in comparison with **2** that has a H-bond. This motion was restricted at 77 K, which led to the higher fluorescence response of **3** at the low temperature.

The fluorescence of frozen **3** exhibited the emission peak of λ_em_ = 563 nm at low temperature (77 K), which was significantly blue, shifted from λ_em_ = 640 nm when observed at 298 K. Interestingly, only one emission peak was detected at 77 K, which could be attributed to emission without ICT interaction. Clearly, the change in temperature (from 285 K to 77 K) caused a notable spectral shift, which was estimated to be Δλ_em_ ≈ 640–563 = 77 nm from **3**, and was comparable to that from **2** (Δλ_em_ ≈ 667–580 = 87 nm). The result thus indicated that the fluorescence signal of **2** at 580 nm (Figure 2b) could be associated with ICT interaction.

Both **2** and **3** have two vinyl bonds that are in *trans-*configuration, as shown by their ^1^H NMR spectra (ESI Figures S1 and S2). It is well known that the vinyl bonds can undergo rapid *cis–trans* photoisomerization upon irradiation in solutions. Such photoisomerization was prevented when the fluorophores were frozen in the solvent matrix. However, the fluorescence intensity of the fluorophores was increased significantly, as the rigid environment at liquid N_2_ temperature reduced the non-radiative decay.

#### 2.2.2. Al^3+^ Chelation

**Scheme 4:**
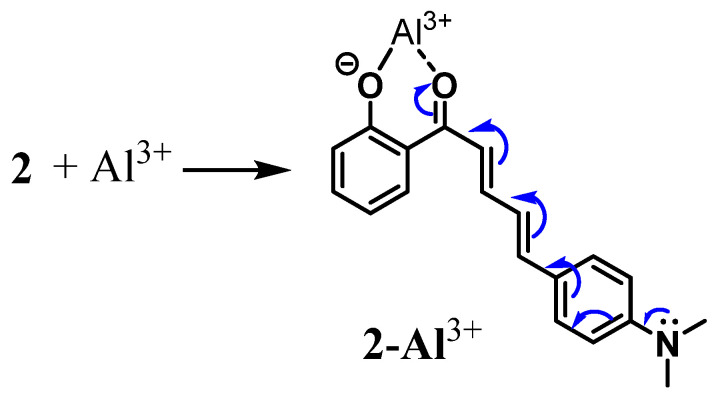
Proposed formation of the Al^3+^ complex **2-Al^3+^**, where the curved arrows show enhanced ICT.

Although aluminum is known for binding 2-hydroxy chalcone ligand **1** [7,8], there is no report found for using chalcone **2**. We therefore sought to synthesize the aluminum complex **2-Al^3+^** (Scheme 4) in order to evaluate its optical properties. In the aluminum complex, the Al^3+^ cation (a Lewis acid) is connected to the carbonyl group, thereby enhancing the ICT interaction between the electron donor (i.e., -NMe_2_) and acceptor (carbonyl) group (Scheme 4). In addition, phenolic hydrogen will no longer to be available in the aluminum complex **2-Al^3+^**, which eliminates the ESIPT pathway.

**Figure 4:**
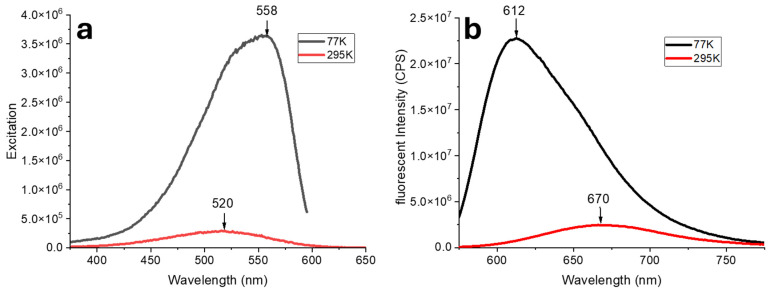
Excitation (**a**) and emission (**b**) spectra of probe–metal complex (**2**-Al^3+^, 10 μM) in ethanol at various temperatures.

Aluminum perchlorate was chosen as the source of Al^3+^ since it had the fastest chelation time among all the aluminum salts tested with **2** (~15 min). At room temperature, the absorption (λ_max_ ≈ 520 nm, ESI S4) and excitation (λ_ex_ ≈ 520 nm) of **2-Al^3+^** (Figure 4a) was red-shifted from that of **2** (λ_ex_ ≈ 480 nm, Figure 2a), as the aromatic phenol (Ar-OH) was converted to phenoxide (Ar-O^−^) in the aluminum complex. At room temperature, formation of the complex did not cause a notable shift in fluorescence (λ_em_ ≈ 670 nm) in comparison with λ_em_ ≈ 663 nm for **2** (Figure 2b). The formation of complex **2-Al^3+^** also led to an appreciable increase in fluorescent intensity (by roughly 5x). The increased fluorescent intensity could be attributed to Al^3+^ chelation that forms a rigid six-membered ring to prevent the free rotation of the phenol ring, which is one of the main nonradiative decay pathways of 2-hydroxy chalcones systems in solution.

Upon freezing to 77 K, the fluorescence of complex **2-Al^3^**^+^ revealed only one peak, with λ_em_ ≈ 612 nm, which was attributed to the emission without ICT. The spectral blue shifted from the fluorescence at temperature 298 K (λ_em_ ≈ 670 nm) and at 77 K (λ_em_ ≈ 612 nm) was estimated to be Δλ_em_ ≈ 670–612 = 58 nm, which was smaller than that for the ligand **2** (Δλ_em_ ≈ 667–580 = 87 nm). On the basis of the large spectral response in responding to low temperature, the emission at 670 nm was assumed to involve ICT interaction. The results from both structurally similar chalcones (i.e., **3** and **2**-Al^3+^) consistently demonstrated that the ICT-based fluorescence should exhibit a large spectral shift when the molecules are frozen in a solvent matrix.

### 2.3. Computational Results

The ground state geometries of **2** and **3** were optimized using Gaussian 09 software, showing a nearly perfect linear cinnamoyl backbone (ESI Figure S5). The intraplanar angle between the A-ring and cinnamoyl backbone was found to be ~42.02° for **2** and ~63.92 for **3**, respectively. The smaller intraplanar angle of **2** (45.02°) could be attributed to the intramolecular H-bonding. Interestingly, the LUMO of **2** revealed significantly higher electron density on the A-ring (ESI Figure S6), in sharp contrast to the LUMO of **3**, whose A-ring showed very low electron density. Significant flow of electron density to the A-ring in the LUMO of **2** could not be explained by considering the ICT only, as shown in Scheme 3. The results thus pointed to the *co*-existence of ESIPT in the excited state, which would permit the electron flow to the A-ring, as indicated by the *keto* tautomer of **2**.

## 3. Materials and Methods

All chemicals were purchased from Sigma Aldrich and TCI America and used without further purification. UV–Vis spectral analysis was performed using a Hewlett Packard-8453 diode array spectrophotometer (Palo Alto, CA, USA) at 25 °C. Fluorescence spectra were measured using a HORIBA Fluoromax-4 spectrofluorometer (Kyoto, Japan). All spectroscopic analysis was performed with a dye concentration of 10 μM unless stated otherwise. ^1^H NMR spectra were obtained on a Varian 500 MHz spectrometer (Palo Alto, CA, USA) in deuterated chloroform.

### 3.1. Synthesis

The synthesis of chalcones **2** and **3** was achieved via Claisen–Schmidt condensation, according to previously reported literature, which is shown in Scheme 2 [13]. The precipitation of **2** occurred slowly over multiple hours after acidifying to pH 2, whereas **3** precipitated out immediately and was isolated in higher yields: 77% vs. 68%, respectively.

#### 3.1.1. Synthesis of (2E,4E)-5-(4-(Dimethylamino)Phenyl)-1-(2-Hydroxyphenyl)Penta-2,4-Dien-1-One (**2**)

Hydroxy chalcone **2** was synthesized according to previously reported methods [13]. To a 50 mL round bottom flask, 0.1 g of NaOH dissolved in 10 mL of MeOH was added, followed by 1 mmol of 4-dimethylamino cinnamaldehyde and 1 mmol of 2-hydroxy acetophenone. The stirring solution was slowly heated up to reflux temperature and refluxed overnight (18 h). After refluxing, the dark red solution was cooled to 25 °C and then poured into 125 mL of ice water with continuous stirring. The red solution was placed onto an ice bath and then neutralized to pH 2 with 6 M HCl dropwise. Precipitation of the product occurred slowly over 24 h. The black precipitate was recrystallized in EtOH to yield dark purple needle-like crystals. Yield: 68%. Melting point 150–151 °C ^1^H NMR (CDCl_3_, 500 MHz) δ 13.10 (br. s, 1H, Ar-OH), 7.86 (d, *J* = 10 Hz, 1H, Ar-H), 7.78-7.73 (dd, *J* = 10 Hz, 15 Hz, 1H, vinyl-H), 7.46 (t, *J* = 10 Hz, 1H, Ar-H), 7.43 (d, *J* = 5 Hz, 2H, Ar-H), 7.11 (d, *J* = 15 Hz, 1H, vinyl-H), 7.03 (d, *J* = 15 Hz, 1H, vinyl-H), 7.01 (d, *J* = 5 Hz, 1H, Ar-H), 6.91 (t, *J* = 10 Hz, 1H, Ar-H), 6.91–6.86 (dd, *J* = 10 Hz, 15 Hz, 1H, vinyl-H), 6.70 (d, *J* = 5 Hz, 2H, Ar-H), and 3.04 (s, 6H, -N(CH_3_)_2_).

#### 3.1.2. Synthesis of (2E,4E)-5-(4-(Dimethylamino)Phenyl)-1-(2-Methoxyphenyl)Penta-2,4-Dien-1-One (**3**)

Methoxy chalcone **3** was synthesized and isolated as described above for hydroxy chalcone **2**. Instead of 2-hydroxy acetophenone, 2-Methoxy acetophenone was used. Precipitation of the product occurred during the course of the reaction. After acidification to ~pH 2, precipitation occurred within 1 h. The red precipitate was recrystallized in EtOH to yield lustrous gold plates. Yield: 77%. ^1^H NMR data are consistent with previously reported data [15].

^1^H NMR (CDCl_3_, 500 MHz) δ 7.55 (dd, *J* = 7.5, 1.8 Hz, 1H, Ar-H), 7.45–7.37 (m, 4H), 7.02 (td, *J* = 7.5, 1.1 Hz, 1H, Ar-H), 6.98 (d, 8.1 Hz, 1H), 6.875 (d, *J* = 15.5 Hz, 1H, vinyl-H), 6.83–6.80 (d, *J* = 15.5 Hz, 1H, vinyl-H), 6.81–6.78 (d, *J* = 15.1 Hz, 1H), 6.67 (d, *J* = 10 Hz, 2H, ArH), 3.88 (s, 3H, -OCH_3_), and 3.00 (s, 6H, -N(CH_3_)_2_).

#### 3.1.3. Fluorescence Quantum Yield Calculation

The fluorescence quantum yield (ϕ_fl_) was calculated using rhodamine 6G as the standard (ϕ_fl_ = 0.95, EtOH) at 488 nm, according to the previously reported procedure [16,17,18]. The following equation was used for calculation. Where Abs is the absorbance of the sample, I is the integrated fluorescence emission intensity, and η is the refractive index of the solvent.

$${(\phi }_{fl})sample=\phi_{Ref}\times \frac{{Abs}_{Ref}}{{Abs}_{Sample}}\times \frac{I_{Ref}}{I_{Sample}}\times \frac{{\left(\eta_{Ref}\right)}^{2}}{{\left(\eta_{Sample}\right)}^{2}}$$

#### 3.1.4. Computational Details

The ground state geometries of chalcones **2** and **3** were optimized using density functional theory (DFT) B3LYP and the 6-31G (d) basis set on Gaussian 09 software. Vertical excitation energies were calculated using TD-SCF, DFT B3LYP, and the 6-31G (d) basis set solving for 10 states (energies). The emission spectrum was calculated using the same parameters that were used for excitation. Excitation and emission parameters were performed using the polarizable continuum model (PCM), with dichloromethane as the solvent. Interplanar angles of the cinnamoyl and the acetophenone fragment were measured using the ground state optimized geometry in the Mercury software (version 2024 1.0).

## 4. Conclusions

The spectroscopic study has shown that chalcone **2** with extended conjugation could also exhibit the excited state intramolecular proton transfer (ESIPT). Although chalcone **2** revealed only one broad emission peak at ~667 nm at room temperature, its solution showed two emission peaks (λ_em_ = 580 and 636 nm) when cooling down to liquid N_2_ temperature (77 K). By freezing molecules to a solvent matrix, molecular motions were prevented in the excited state, thus eliminating possible transitions such as TICT. This could reveal the potential hidden transition(s) that are difficult to observe at room temperature. With the aid of the methoxy chalcone **3**, one of the emission peaks (e.g., λ_em_ = 580 nm) could be assumed to be associated with ICT, or locally excited (LE) state without ICT. This was further confirmed by using the aluminum complex **2**-Al^3+^, which also removed the impact of ESIPT. Thus, the major emission peak (λ_em_ = 636 nm) of **2**, observed at low temperature, could be attributed to ESIPT.

It appeared that the ICT- and ESIPT-associated fluorescence signals were coupled into one fluorescence peak for chalcone **2**. The computational study also indicated a high electron density on the A-ring in the excited state of **2**, suggesting the significant involvement of ESIPT in the emission. The study also illustrated that separation of ICT from ESIPT required a very rigid environment. This was consistent with the less conjugated 2′-hydroxychalcone **1**, whose ESIPT can only be observed in a more rigid environment. The finding thus adds some knowledge to the photophysical properties of chalcones with extended conjugation. These results could serve as a useful guide for further development of chalcone based optical materials.

## Figures and Tables

**Table 1:** Spectroscopic properties of chalcones **2** and **3** in different solvents.

Compound	Solvent	λ_abs_ (nm)	λ_Em_ (nm)	Φ_fl_	ε (M^−1^cm^−1^)	Δλ (cm^−1^)
**2**	Hexane	431	*	**	15,818	-
	Toluene	452	550	**	47,874	3942
	DCM	461	628	0.003	45,593	5768
	EtOH	454	665	0.016	45,412	6989
	EtOAc	449	600	0.001	48,398	5605
	MeOH	453	680	0.010	44,627	7369
	ACN	456	655	0.015	46,355	6663
	DMF	468	662	0.066	44,163	6262
	DMSO	471	663	0.032	42,052	6148
	H_2_O	458	*	0.002	22,685	-
	EtOH•Al^3+^	512	670	0.062	34,614	4606
**3**	Hexane	398	*	**	2238	-
	Toluene	415	510	0.019	41,370	4489
	DCM	424	580	0.063	32,640	6344
	EtOH	429	640	0.128	44,492	7685
	EtOAc	410	547	0.050	48,632	6109
	MeOH	430	650	0.041	41,916	7871
	ACN	418	601	0.160	42,406	7285
	DMF	426	604	0.204	47,226	6918
	DMSO	433	621	0.283	44,010	6992
	H_2_O	435	635	0.008	29,790	7240

* Nonfluorescent, ** Fluorescence was too weak to accurately calculate quantum yield.

## Data Availability

Data is contained within the article and Supplementary Materials.

## Supplementary Materials

The following supporting information can be downloaded at: https://www.mdpi.com/article/10.3390/molecules29245972/s1.
