# Supplementary material for: Fluorescence of 2-Hydroxy Chalcone Analogs with Extended Conjugation: ESIPT vs. ICT Pathways
Source: Molecules. 2024 Dec 18;29(24):5972. doi: 10.3390/molecules29245972 (PMC11677836; doi:10.3390/molecules29245972)
Supplement: Supplementary file 1 [file molecules-29-05972-s001.zip › molecules-3355087-supplementary.pdf]

Supplementary Information

for

Fluorescence of 2-Hydroxy Chalcone Analogues with Extended Conjugation: ESIPT vs ICT Pathways

Brian Corbin , Paityn Houglan , Yi Pang*

Department of Chemistry, The University of Akron, Akron, Ohio 44325

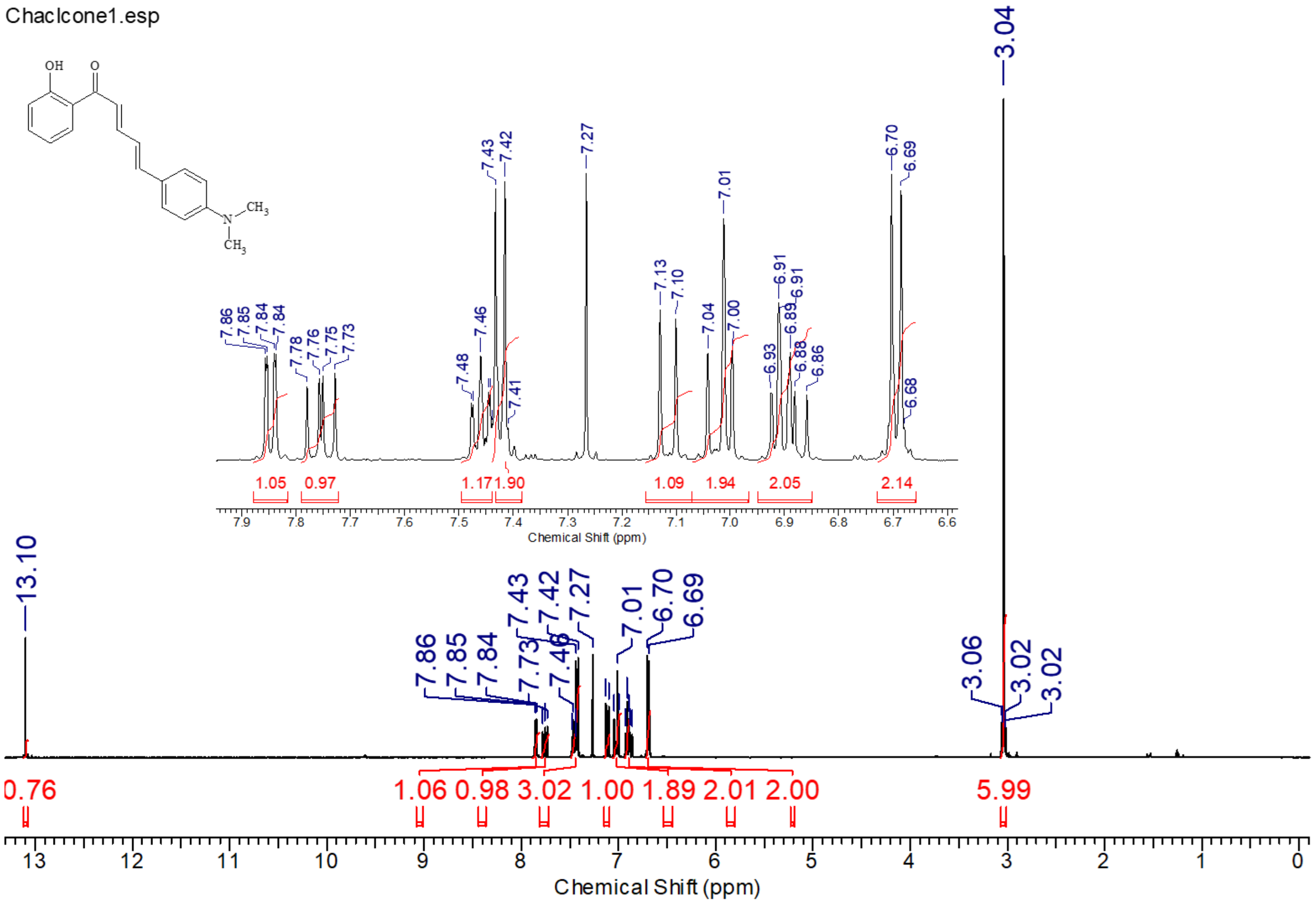

S1a 500 MHz ^{1}HNMR of chalcone **2** in $CDCl_3$.

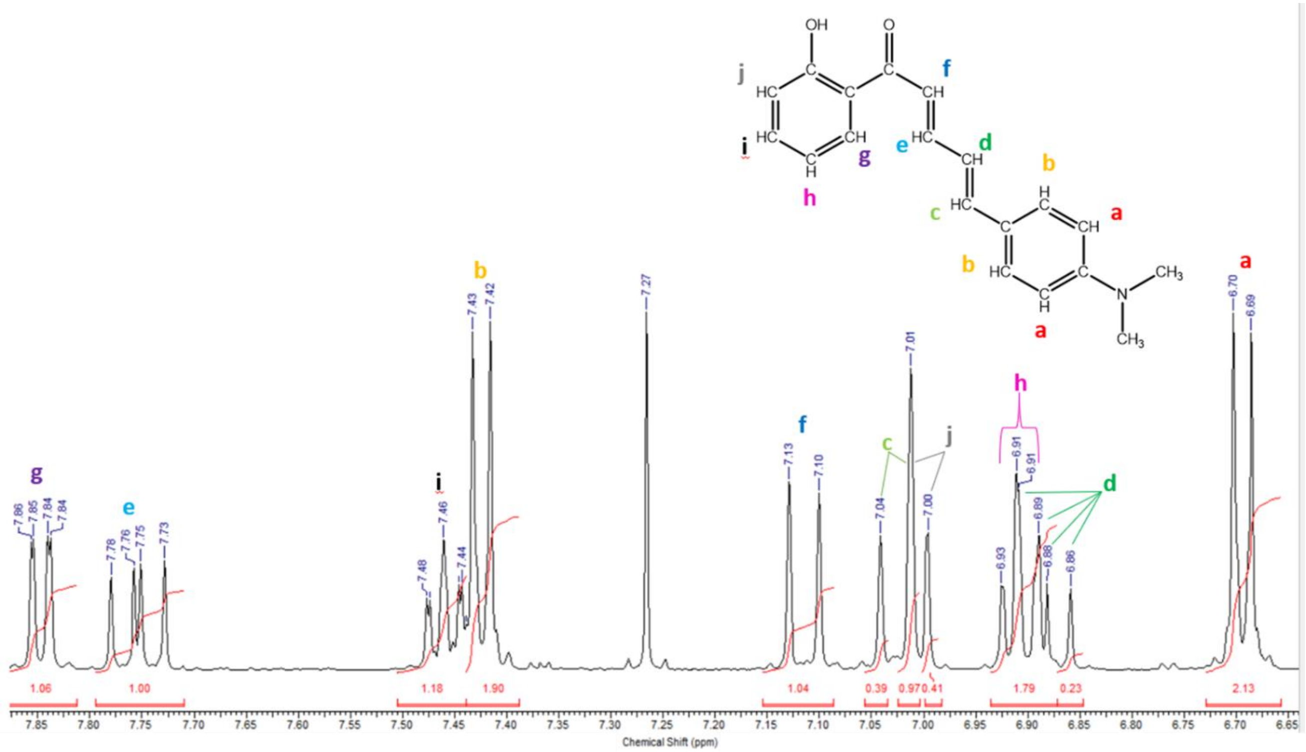

S1b 500 MHz ^{1}HNMR of chalcone **2** in $CDCl_3$. Expanded aromatic region of **2** with assigned aromatic hydrogens

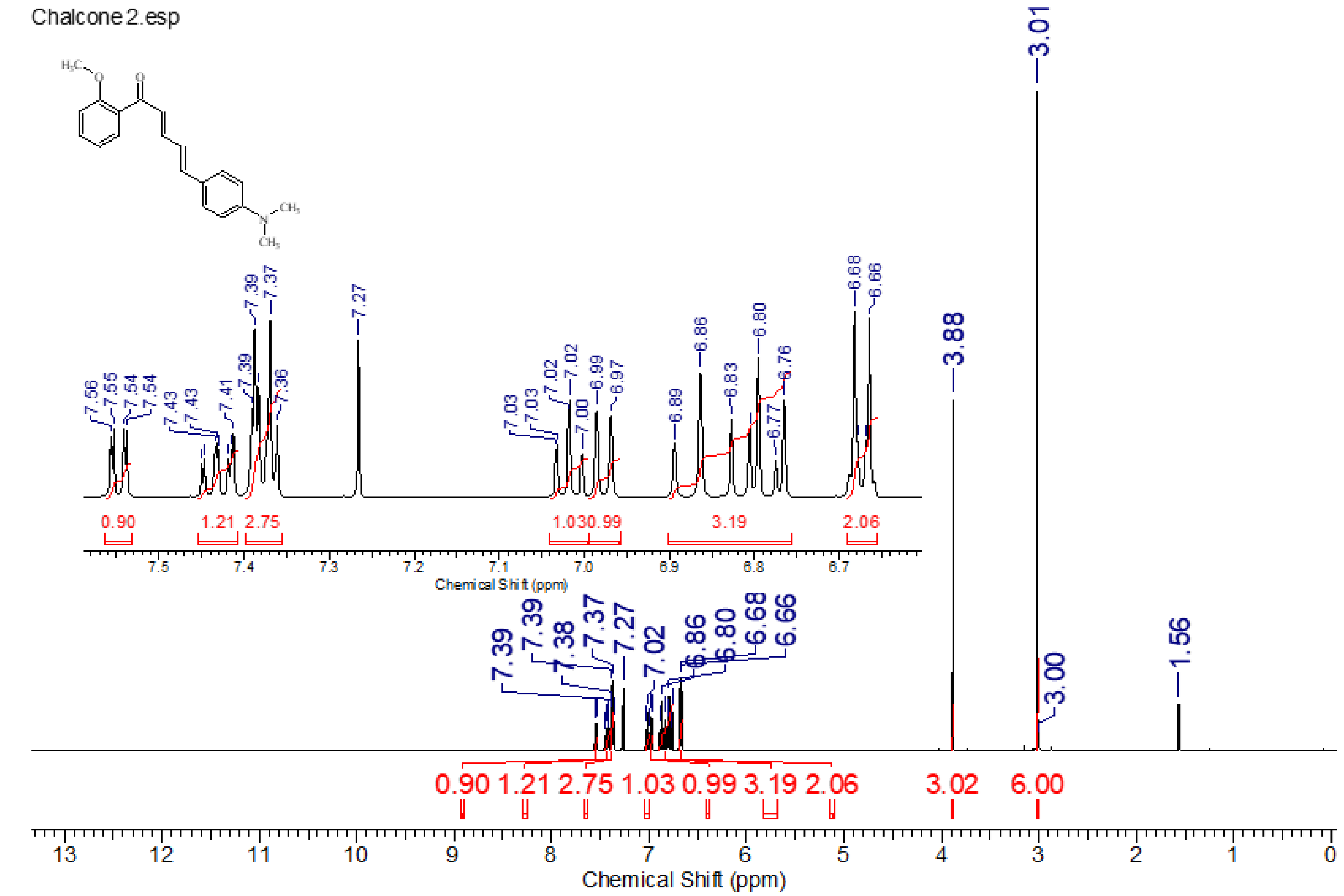

S2 500 MHz ^{1}H NMR of chalcone **2** in $CDCl_3$. The inset is a zoomed in view of the aromatic region.

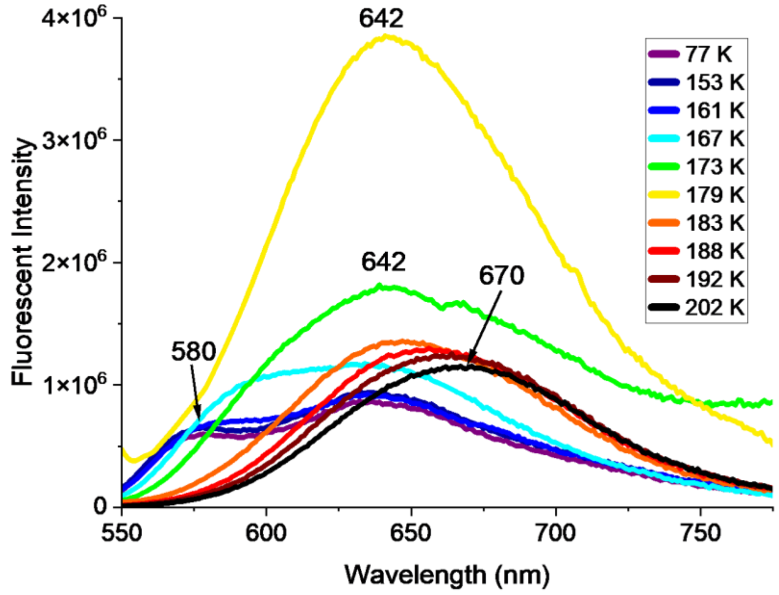

S3 Low temperature fluorescence of chalcone **2** in EtOH at various temperatures.

Al^{3+} chelation procedure

First, a stock solution of 10 mM Al(ClO$_4$) • 9 H_2O was freshly prepared. 2 mL of 100% EtOH was added into a quartz cuvette followed by 2 µL of **2** from a 10 mM DMSO stock solution to achieve a final dye concentration of 10 µM. The solution was mixed thoroughly via inversion and then 3 equivalents of Al^{3+} (6 µL of 10 mM stock) were added and mixed by inversion. The solution was rested for 15 minutes before any testing, during which, there is a noticeable color change from yellow to purple of the ethanol solution indicating chelation of aluminum to the dye has occurred. 3 equivalents of aluminum were used to ensure there was no free chalcone in solution. Therefore, all spectroscopic results will be from the chalcone-metal complex.

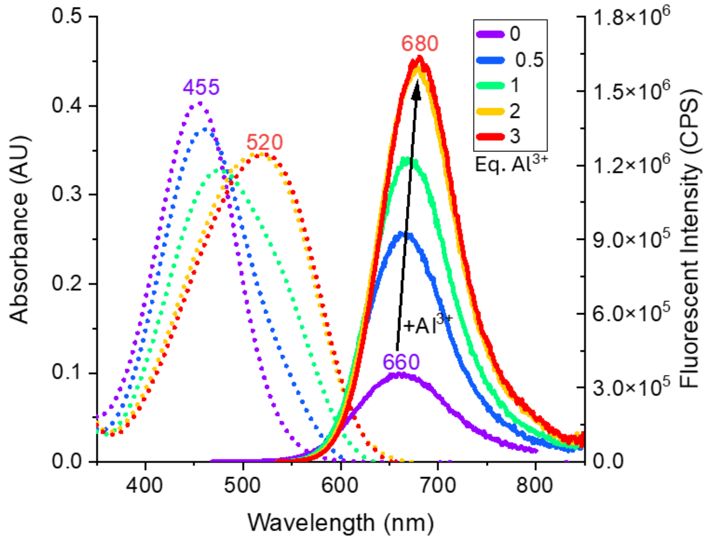

S4 Titration of **2** with aluminum perchlorate in EtOH at room temperature. Absorbance is shown as dotted lines and emission as solid lines. Incubation period of 15 minutes for all samples.

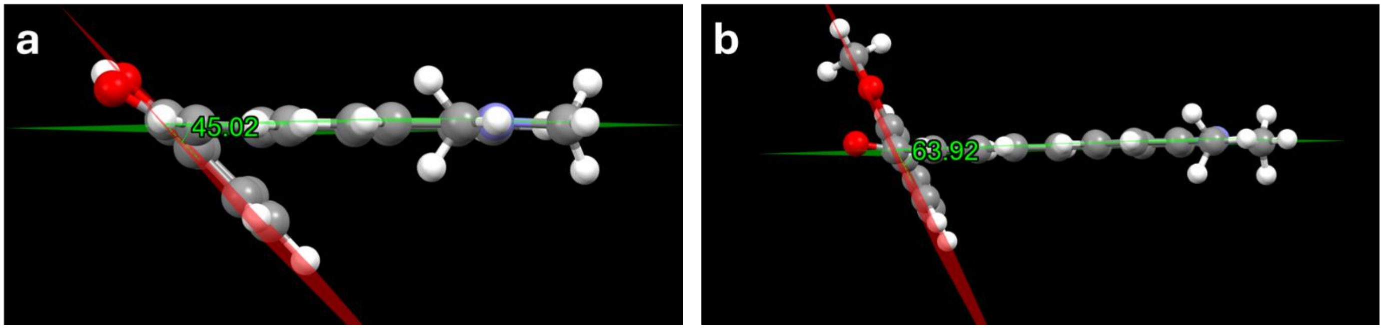

S5 Optimized ground state intraplanar angles of the A-ring (red) vs cinnamoyl backbone (green) of **2** (a) and **3** (b). Geometry optimization performed using DFT B3LYP, 6-31G (d) basis set on Gaussian 09 software.

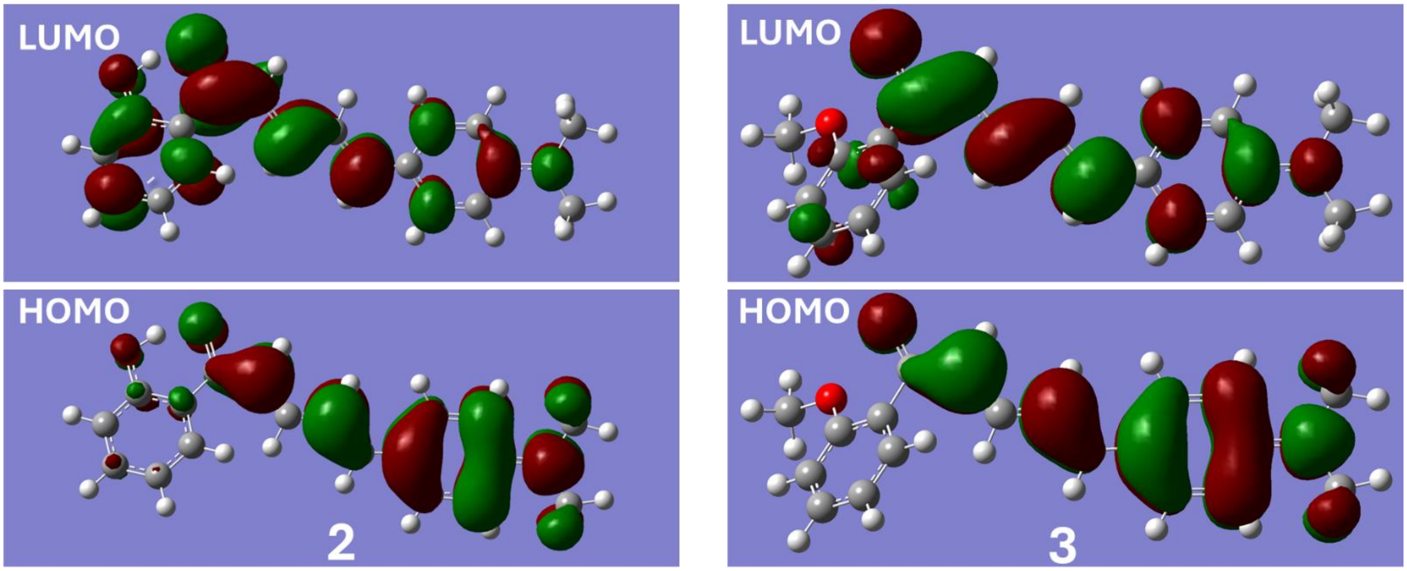

S6 Frontier molecular orbital diagram for **2** (left) and **3** (right).

S7 Select DFT data from computational studies of chalcones **2** and **3**. The absorbance and emission were calculated using the optimized S_0 and S_1 geometry respectively. Oscillator strength (f) is provided for absorbance and emission data. The change in dipole moment (Δμ) is calculated as $\mu S_1 - \mu S_0$ and given in units of Debye (D).

**Compound**	λ_{abs} nm	**f**	λ_{em} nm	**f**	**Dipole (**μ) S_0	**Dipole (**μ) S_1	Δμ (D)
2	464	1.165	557	1.47	9.45	15.44	5.99
3	428	1.31	496	1.57	7.52	12.03	4.51
